# The Threat of Antibiotic Resistance in Changing Climate

**DOI:** 10.3390/microorganisms8050748

**Published:** 2020-05-16

**Authors:** Aliyar Cyrus Fouladkhah, Brian Thompson, Janey Smith Camp

**Affiliations:** 1 Public Health Microbiology Laboratory, Tennessee State University, Nashville, TN 37209, USA; 2 School of Public Health, Yale University, 60 College St, New Haven, CT 06510, USA; brian.thompson@yale.edu; 3 Department of Civil and Environmental Engineering, Vanderbilt University, Nashville, TN 37235, USA; janey.camp@vanderbilt.edu

As the earliest form of life, microorganisms have elaborate mechanisms for adapting to changes in environmental conditions. Vertical and horizontal gene transfer mechanisms, formation of sessile biofilm communities, and cell-to-cell communication via quorum sensing enable microbial populations to efficiently move towards diversity and fitness [1]. Anthropogenic emissions of greenhouse gasses have forced post-industrial warming of climate, which has resulted in each of the last three decades being warmest on record [2], and as of 2017, global temperatures had risen by over ~1 °C from pre-industrial levels [3]. Without mitigation efforts, global temperature is projected to rise by as much as 4.8 °C by the end of 21st century [2]. At our current rate, we could expect to cross the 1.5 °C global temperature increase threshold by 2040 [3]. Rates and effects of climate change are globally non-uniform—the Arctic may warm by as much as 11 °C [2].

Spread of infectious diseases in landscapes of changing climate cannot inherently be characterized with complete certainty, due to the complexity and diversity of microbial populations. Nevertheless, it is well established that increases in environmental temperatures appreciably augment multiplication of an array of infectious disease agents. It is estimated that a 1 °C increase in environmental temperatures (above 5 °C) could lead to 5%–10% increases in cases of salmonellosis [4], a disease that is estimated to cause more than one million episodes of foodborne illness annually in the United States (27.2% hospitalization rate) [5]. A 1 °C temperature increase could cause an additional 50,000 to 100,000 foodborne salmonellosis illness episodes and an additional 27,000 annual hospitalizations in the United States. This estimated additional burden is only for one pathogen, in one region of world, and only for a 1 °C increase in environmental temperature. There are >200 known diseases that are transmitted through contaminated food and water across the globe, with many requiring antibiotic treatment.

In addition to vector-borne, foodborne, and zoonotic infectious diseases, since the 1854 cholera outbreak in Soho, London, which served as the cornerstone of modern epidemiology, the spread of infectious diseases in surface and sub-surface waters has been a persisting public health challenge. Modelling studies on climate change-induced spread of *Vibrio cholerae* well articulate the magnitude of waterborne diseases in the landscape of changing climate. Modeling projections for coastal waters conducive to multiplication of *Vibrio cholerae* under conservative climatic change estimates illustrate a potential increase in areas with already vulnerable populations and shifts northward to areas where suitable conditions may exist in future [6]. This indicates that areas already at risk may have more cases of cholera to manage and that areas not yet affected may need to prepare [6]. As stated by Lemery and Auerbach [7], “Climate change perhaps will not create new diseases, but it will broadly increase the human populations at risk, and thereby make the imperative for effective vaccines and protective guidance more urgent”.

While treatments for the vast majority of bacterial infections heavily rely on the use of antibiotics, the efficacy of these treatments is diminishing, with 18 pathogens currently listed as “urgent”, “serious” and, or “concerning” public health threats in the United States for developing antibiotic resistance [8]. The high rates of this concern have similarly been reported in all regions of the World Health Organization [9]. Extensive use of antibiotics, such as sub-therapeutic utilization in animal industry or prolonged administration to patients, and to a lesser extent intense utilization, are main contributors to antibiotic resistance concern [10,11]. It is noteworthy that, due to the complexity of the animal husbandry environment, the direct impacts of sub-therapeutic and prophylactic use of antibiotics and antimicrobial compounds in the food animal industry on development of antibiotic resistance threat in humans and clinical settings are not completely well-characterized. Nevertheless, around one out of five resistant infections in humans are derived from food and animals [11]. The United States currently experiences >2.8M episodes of antibiotic-resistant human infections, leading to at least 35,000 annual deaths in a typical year [8]. The current economic burden of antibiotic resistance in public health settings without implementing control measures is around USD 7 billion annually in the United States [11]. Implementing best practices such as vaccination, optimized nutrition, and preventive infection biosecurity measures could be feasible alternatives to sub-therapeutic and prophylactic administration of antibiotics in animal husbandry for mitigating the risk of this potential and important route of resistant infections in humans [8,11].

Given the wide-spread and expanding nature of the antimicrobial resistance threat, considerable difficulties in identification and regulatory approval of new classes of antibiotics, the tremendous ability of bacterial pathogens to adapt to an antibiotic, and that climate change is a “threat multiplier” for spread of infectious diseases and antibiotic resistance, relying on the discovery of new antibiotics alone cannot be the sole efficacious and sustainable strategy for addressing this public health challenge. Climate change mitigation efforts require collective endeavors of governmental and non-governmental agencies, private industries, and consumers. In addition to these efforts, climate-smart and evidence-based adaption and resilience programs could further assure prevention of premature morbidity and mortality associated with climate-change-induced antibiotic resistance and infectious diseases. With substantial use of therapeutic and sub-therapeutic antibiotics in food animal husbandry, any vulnerability assessment, evidence-based intervention, and stewardship program will have to embrace the “one health” approach. This approach should prioritize limiting the extensive use and prophylactic and sub-therapeutic administration of antibiotics in humans and in animal husbandry, instead of limiting the intense use of antibiotics by individuals in need of a treatment in a healthcare facility. The climate-change-induced antibiotic resistance threat will disproportionally affect citizens of countries with suboptimal public health infrastructure—those who have contributed least to climate change. This will be a pronounced public health challenge for all age groups and particularly for vulnerable populations including the very young, elderly, immunocompromised, and the pregnant women.

The current Special Issue publishes recent advancements in mitigation and/or elimination of foodborne and waterborne microorganisms and antibiotic resistance. Such information will be of utmost importance for assuring the safety of our food and water supplies and for conduct of vulnerability assessments and development of mitigation, adaption, and resilience programs in the landscape of our changing climate. Special emphasis is placed on publications of emerging, efficacious, and economically feasible technologies for control of serovars of non-typhoidal *Salmonella enterica*, various serogroups of Shiga toxin-producing *Escherichia coli*, public health significant serotypes of *Listeria monocytogenes*, pathogenic *Vibrio* species, drug-resistant *S. aureus*, *Campylobacter* spp., pathogenic *Cronobacter* spp., and norovirus. Researchers and practitioners conducting original laboratory studies with sessile and planktonic microorganisms, and those conducting risk assessment analyses, epidemiological research, and critical and systematic reviews of literature, are cordially invited to submit a manuscript for this Special Issue of *Microorganisms*.
